# Prevention of surgical site infection: a ten-step approach

**DOI:** 10.1186/s42836-023-00174-7

**Published:** 2023-04-08

**Authors:** Saad Tarabichi, Javad Parvizi

**Affiliations:** Rothman Orthopaedic Institute at Thomas Jefferson University Hospital, Philadelphia, PA 19107 USA

**Keywords:** Total Joint Arthroplasty, Surgical Site Infection, Periprosthetic Joint Infection, Prevention

## Abstract

Surgical site infection (SSI) is a common cause of morbidity and mortality in patients undergoing surgery. Similarly, periprosthetic joint infection (PJI), is a major cause of failure after total joint arthroplasty (TJA). As the annual volume of TJA procedures is projected to rise, so will the rate of subsequent SSI and PJI. Currently, prevention has been identified as the single most important strategy for combating SSI/PJI. Hence, the present article will serve as a summary of an evidence-based ten-step approach for SSI/PJI prevention that may help orthopedic surgeons with their infection prevention strategies.

## Background

Despite global efforts, surgical site infection (SSI) remains a significant cause of morbidity and mortality in surgical patients [1]. In a recent study, the American College of Surgeons estimated that SSI cost the United States healthcare system between $3.5 to $10 billion in 2016 alone [2]. Similarly, periprosthetic joint infection (PJI) is a catastrophic complication and a major cause of failure after primary and revision total joint arthroplasty (TJA) [3]. As the number of TJA procedures performed annually continues to increase, so will the rate of subsequent PJI [4].

To date, prevention has been identified as the single most important strategy in combating SSI/PJI [5]. Recent clinical practice guidelines (CPGs) on infection prevention and control have identified a host of modifiable risk factors that can help mitigate the risk of SSI/PJI [6, 7]. Of note, this article will discuss preventive practices currently endorsed by the most recent CPGs on SSI/PJI prevention from the following organizations: (1) Centers for Disease Control and Prevention, (2) International Consensus Meeting on Musculoskeletal Infection, and (3) the American Academy of Orthopaedic Surgeons [8–10]. Traditionally, prevention in this setting has highlighted two main groups of risk factors: (1) patient-specific risk factors, and (2) environmental risk factors. However, given the multifactorial etiology of these disease processes, we believe a more holistic approach is warranted and have thus chosen to report the most important steps in SSI/PJI prevention (Table 1).

**Table 1:** Ten steps of SSI/PJI prevention

**Step 1**	Optimization of host risk factors
**Step 2**	Bioburden reduction
**Step 3**	Perioperative antibiotic prophylaxis
**Step 4**	Respect for soft tissues
**Step 5**	Expeditious surgery
**Step 6**	Minimization of blood loss
**Step 7**	Reducing operating room traffic
**Step 8**	Antiseptic irrigation solution
**Step 9**	Sterilization of implants & instruments
**Step 10**	Wound management

This article will serve as a brief summary of ten practical and effective measures currently employed to help prevent SSI/PJI development after TJA at our institution.

### Host optimization

Recently, CPGs on the prevention of SSI have placed great emphasis on preoperative optimization and risk stratification of patients undergoing TJA [8]. In addition to known absolute contradictions to surgery, such as the presence of an active infectious lesion near the intended incision site, a number of modifiable host risk factors have also been identified [10]. These include, but are not limited to, diabetes, hypertension, malnutrition, immunocompromised state, high body mass index (BMI), history of smoking, corticosteroid use, and malnutrition [11]. In particular, preoperative hyperglycemia is one such risk factor that is increasingly prevalent in this patient population, with one study demonstrating that > 30% of seemingly "healthy" patients undergoing TJA had undiagnosed hyperglycemia [12, 13]. Although a growing body of evidence has suggested that HbA1c may not be as reliable for assessing glycemic control as previously believed, it remains the "gold standard" index for identifying poor glycemic control in patients undergoing surgery [14, 15]. Current recommendations from American Diabetes Association dictate that surgery be delayed in all patients with an HbA1c > 7% [16]. More recently, fructosamine has been identified as a promising marker for predicting outcomes in patients undergoing TJA [17, 18]. A multicenter study found that fructosamine was superior to HbA1c in predicting adverse outcomes following TJA [19].

### Reducing bioburden

The use of preoperative skin preparations to reduce host bioburden is increasingly common and has been adopted worldwide [20]. The rationale behind this strategy is that effective skin decontamination, along with the removal of hair near the surgical site, can cause a significant reduction in the number of bacteria [9]. However, it is important to note that aggressive application of skin preparations may inadvertently result in damage to superficial skin layers and thus paradoxically increase the risk of infection. In addition to the perioperative treatment of the intended incision site with antiseptic agents, current CPGs recommend all patients undergoing TJA bathe with antiseptic soap in the days leading up to their procedure [9]. Furthermore, nasal colonization with *Staphylococcus aureus* has also been shown to increase the risk of SSI [21]. Although several organizations have endorsed preoperative screening or universal decolonization to mitigate the risk of *Staphylococcus aureus* nasal carriage, neither protocol has proven effective at reducing SSI rates [22, 23].

### Perioperative antibiotic prophylaxis

Perioperative antibiotic prophylaxis for patients undergoing TJA is a proven method of SSI prevention [24]. Due to their relatively benign risk profile and broad-spectrum antimicrobial activity, recent CPGs have endorsed the use of either first or second-generation cephalosporins as the primary method of antibiotic prophylaxis in this setting [25]. Although surgeons have traditionally been discouraged from administering cephalosporins in patients with self-reported penicillin allergies, we now know that cross-reactivity between cephalosporins and penicillin is uncommon [26]. Furthermore, in the absence of a documented history of an anaphylactic reaction to penicillin, self-reported penicillin allergies are often inconsequential, making cephalosporins a safe and practical option in these patients [27]. It is also recognized that in order to obtain optimum antibiotic coverage, a single weight-adjusted (15 mg/kg) dose of antibiotic prophylaxis must be administered 30 to 60 min prior to skin incision [25, 28]. However, certain high-risk patients may require additional antibiotic coverage. For example, the use of dual antibiotic prophylaxis, consisting of cephalosporin and vancomycin, is warranted in all patients considered at high risk of developing a methicillin-resistant *Staphylococcus aureus* infection [29]. Due to its prolonged infusion time, administration of vancomycin should begin 60–90 min prior to skin incision [30].

### Respect for soft tissues

Respect for soft tissues is a commonly overlooked mode of SSI prevention. It is vital to ensure that soft tissues are properly handled by instruments and not by hand and potentially contaminated gloves. In addition, excessive tension on the skin and soft tissues should be avoided. A small but adequate incision size should be used to conduct the surgery safely. In practice, this is done by ensuring that the incision size is large enough to allow for proper anatomic visualization of the joint and insertion of implant components. Furthermore, the utilization of non-absorbable sutures and the liberal use of electrocautery have both been shown to increase the risk of infection [31, 32].

### Expeditious surgery

Protracted operative time is a well-established risk factor for SSI development. A recent study found that the likelihood of SSI increased by 37% for every 60 min of surgery [33]. Similarly, it has been shown that a 20-min increase in operative time can increase the risk of PJI by as much as 25% [34]. While the exact mechanism behind this remains unclear, it is postulated that the longer the procedure, the higher the chance of surgical field contamination [35, 36]. In addition, due to the relatively short half-life of commonly used antimicrobial prophylaxis, antibiotic tissue penetration can drop off significantly during longer procedures if the prophylactic agent is not properly re-dosed [37]. Lengthier procedures also lead to prolonged tourniquet use, increasing the potential for local tissue hypoxia [38]. However, it is important to acknowledge that difficult procedures will, inevitably, require longer operative times. Therefore, to ensure that the technical aspects of the procedure are not compromised, efforts to reduce operative time must encompass the safety of the procedure.

### Minimizing blood loss

It is recognized that the need for allogeneic blood transfusions increases the risk of SSI and PJI [11]. Therefore, strategies to prevent unnecessary blood loss during TJA are of great importance. These include but are not limited to the use of hypotensive anesthesia, correction of anemia prior to admission, tranexamic acid administration, and tourniquet use [39]. Of note, perioperative administration of tranexamic has demonstrated excellent efficacy for the reduction of blood loss, the need for allogeneic blood transfusion, and periprosthetic joint infection [40]. In addition to this, local application of topical hemostatic agents has been shown to reduce the risk of bleeding postoperatively [39]. Furthermore, aggressive venous thromboembolism (VTE) prophylactic agents increase the risk of bleeding and should therefore be avoided [41].

### Reducing operating room traffic

Intraoperative wound contamination is commonly brought on by airborne pathogens present in the operating room (OR) [42]. It is also well-established that the majority of airborne pathogens in the OR originate from members of the surgical team. Current guidelines on infection prevention and control recommend that the number of surgical personnel be kept to a minimum, without compromising the patient care [43, 44]. Furthermore, excessive opening/closing of the OR doors should be avoided as it can generate air currents that may increase the chances of surgical field contamination [45].

### Antiseptic irrigation solution

The use of irrigation solutions is paramount to ensure effective chemical and mechanical debridement of tissues [46]. The selection of an appropriate antiseptic irrigation solution is largely up to the preference of individual surgeon. Based on extensive data spanning over many years, 0.5% povidone-iodine (PVP-I) irrigation solution is currently the choice of our institution and many others. The lack of toxicity of the PVP-I to fibroblasts, which has been reported with other antiseptic solutions [47], and proven efficacy has led to the popularity of PVP-I irrigation solutions.

### Cleaning of implants and instruments

Sterilization of implants and surgical instruments prior to surgery is essential to preventing SSI and PJI [48]. Currently, validated methods of sterilization of orthopedic implants and devices include, but are not limited to, radiation, ethylene oxide gas, and vaporized hydrogen peroxide [49]. In addition to this, we now know that intraoperative contamination of implants is common [50]. Recent protocols on infection prevention have endorsed the use of several strategies to reduce the risk of intraoperative implant contamination. These include, but are not limited to, assessing the sterility of surgical tray wraps, minimizing implant exposure to OR air, changing gloves before handling of implants, and ensuring implants do not come in direct contact with patient skin [51]. Of note, the sterility of surgical tray wraps is often compromised. Furthermore, current methods for evaluating surgical tray wraps for breaches are not as reliable as previously believed [52].

### Wound management

Proper wound closure and application of appropriate skin dressing is vital to reducing the risk of SSI [53]. This can be done by suturing in extension following total hip arthroplasty and suturing in ten degrees of flexion following total knee arthroplasty. Furthermore, strong subcutaneous suture lines can help significantly reduce tension on skin sutures and prevent excessive wound tightness. Recently, wound closure using a subcuticular suture and skin adhesive has been shown to reduce the risk of superficial drainage [54]. Furthermore, silver-impregnated occlusive dressings have shown great promise in reducing infection rates in patients undergoing TJA [55]. It is also important to note that less aggressive anticoagulants, such as aspirin, can help significantly reduce wound drainage [56].

## Conclusion

In summary, it is evident that the prevention of SSI and PJI is multifactorial and requires a multidisciplinary approach. Furthermore, as advancements in technology bring forward new methods of prevention in this setting, it is paramount to ensure the validity of these strategies. Notwithstanding, the present article provides a brief summary of a proven ten-step approach for SSI and PJI prevention. Of note, preoperative host optimization, administration of prophylactic antibiotics, use of antiseptic irrigation solutions, and proper wound management are among the most important preventive measures currently available in this setting.

## Data Availability

Not applicable.

## Abbreviations

SSI: Surgical Site Infection
PJI: Periprosthetic Joint Infection
TJA: Total Joint Arthroplasty
CPGs: Clinical Practice Guidelines
BMI: Body Mass Index
VTE: Venous Thromboembolism
OR: Operating Room
PVP-I: Povidone-Iodine
